# Selective Alteration of the Left Arcuate Fasciculus in Two Patients Affected by Creatine Transporter Deficiency

**DOI:** 10.3390/brainsci14040337

**Published:** 2024-03-30

**Authors:** Maurizio Balestrino, Enrico Adriano, Paolo Alessandro Alì, Matteo Pardini

**Affiliations:** 1 Department of Neurosciences, Rehabilitation, Ophthalmology, Genetics and Maternal and Child Sciences (DINOGMI), University of Genoa, 16132 Genoa, Italy; adriano@neurologia.unige.it (E.A.); paoloalessandroali@gmail.com (P.A.A.); matteo.pardini@unige.it (M.P.); 2 IRCCS Ospedale Policlinico San Martino, 16132 Genoa, Italy

**Keywords:** creatine, transporter, deficiency, tractography, magnetic resonance, arcuate fasciculus, arcuate bundle

## Abstract

(1) Background: In hereditary creatine transporter deficiency (CTD), there is an absence of creatine in the brain and neurological symptoms are present, including severe language impairment. However, the pathological changes caused by creatine deficiency that generate neuropsychological symptoms have been poorly studied. (2) Aims: To investigate if the language impairment in CTD is underpinned by possible pathological changes. (3) Methods: We used MRI tractography to investigate the trophism of the left arcuate fasciculus, a white matter bundle connecting Wernicke’s and Broca’s language areas that is specifically relevant for language establishment and maintenance, in two patients (28 and 18 y.o.). (4) Results: The T1 and T2 MRI imaging results were unremarkable, but the left arcuate fasciculus showed a marked decrease in mean fractional anisotropy (FA) compared to healthy controls. In contrast, the FA values in the corticospinal tract were similar to those of healthy controls. Although white matter atrophy has been reported in CTD, this is the first report to show a selective abnormality of the language-relevant arcuate fasciculus, suggesting a possible region-specific impact of creatine deficiency.

## 1. Introduction

Creatine transporter deficiency (OMIM number 300352 [1]) is a rare, hereditary (X-linked) disease that is due to an impairment of the creatine transporter. Without this transporter, creatine cannot cross neither the blood–brain barrier nor the cell plasma barrier, thus it is absent in all brain cells and severe neurological impairment occurs, including defective language development [2]. The neurological symptoms due to the lack of brain creatine are usually attributed to perturbations to energy metabolism. In fact, creatine is phosphorylated by the creatine kinase enzyme, and the creatine–phosphocreatine system is essential in brain energy metabolism [3]. Given this pivotal role of creatine, the research has so far mainly focused on the metabolic and functional effects, and on the neuropsychological symptoms of brain creatine deficiencies [4,5]. In contrast, the pathological and structural basis of such changes have been rarely investigated. Even in the recently developed animal models of creatine transporter deficiency, the possible pathology was not studied; all the investigations focused mostly on the metabolic and neuropsychological changes [6], with the relevant exception of Duran-Trio et al. who found abnormal histological changes in the cerebellum, including reduced synaptic spine density and synaptic spines that were significantly thinner and shorter in the Purkinje neurons of transporter-deficient males [7]. Therefore, it is usually assumed that symptoms are merely functional in nature and prone to disappearance upon restoration of normal creatine levels (this assumption holds for the two creatine deficiency syndromes where creatine supplementation is effective, but in creatine transporter deficiency, such supplementation is useless because no creatine can reach the brain due to the transporter impairment [8]). Nevertheless, it is recognized that even in treatable hereditary creatine deficiency syndromes, replacement therapy with exogenous creatine needs to be administered very early in life for maximal efficacy; for example, in arginine–glycine amidino transferase (AGAT) deficiency, it is only fully effective when creatine is administered starting at birth [9,10]. Creatine administration at later ages is still effective, but the symptoms are not usually fully reversed despite the substantial recovery of brain creatine levels [11]. A possible explanation may be that creatine is implicated in nervous system development, for example, in the differentiation of several cell types, including neurons [12,13]. Thus, the harmful role of brain creatine deficiency may go beyond “functional” energy deficiency to involve structural or histological changes in brain tissue. If this is the case, then we should hypothesize that brain malfunction in creatine transporter deficiency is due not simply to the absence of creatine, but to a deficiency in brain development and possibly wiring. This would carry important implications for possible treatments of this disease, as it would provide further rationale for initiating treatment as early as possible during the period of brain development to minimize the structural damage to the brain due to the lack of creatine.

While histological and postmortem human studies of creatine transporter deficiency are lacking, some morphological data have been obtained with neuroimaging. In the extensive review by van de Kamp et al. of 101 males with X-linked creatine transporter deficiency, brain magnetic resonance imaging (MRI) was available in 76 patients and only showed mild abnormalities in 53 patients. Such abnormalities included mildly delayed myelination, (T2-) hyperintensities, a thin corpus callosum, mildly enlarged ventricles/extracerebral spaces and cerebral/cerebellar atrophy (progressive in two brothers) [14]. Subsequently, Heussinger et al. reported MRI data from two patients with creatine transporter deficiency, which showed different findings despite similar neuropsychological changes, namely brain atrophy in one subject and normal findings in the other one [15].

In the present study, we took advantage of the fact that creatine transporter deficiency includes among its symptoms a prominent language disturbance, similar to aphasia [16,17], and thus decided to study the arcuate fasciculus, a white matter bundle connecting Wernicke’s and Broca’s areas, which are altered in children with language development disorders [18].

## 2. Materials and Methods

### 2.1. Tractography

We used MRI tractography [19] in two patients (28 and 18 y.o., respectively) to investigate if creatine deficiency is accompanied by changes in the arcuate fasciculus of the left hemisphere. We focused on this structure because (1) white matter changes were reported in creatine transporter deficiency and speech delay is a prominent feature of this disease [14], and (2) the arcuate fasciculus of the left hemisphere is critical for all aspects of language, from spontaneous speech and word retrieval to repetition and comprehension abilities [20]. Magnetic Resonance Imaging (MRI) was performed with a 1.5 Tesla scanner (Siemens Medical Solutions) with an eight-channel transmit–receive head coil. The protocol comprised volumetric T1-weighted (T1w) and T2-weighted imaging and High Angular Resolution Diffusion Imaging “HARDI” data as well as single-voxel spectroscopy (MRS) in the left frontal white matter. HARDI was performed using single-shot spin-echo echo-planar imaging (TR/TE/Flip Angle = 13,700 ms/90 ms/90°, field of view = 24 × 24 cm^2^, matrix = 96 × 96 with diffusion gradients applied in 61 non-collinear directions (b value = 1000 s/mm^2^) and six images without diffusion gradients).

The data were analyzed using the FMRIB [Functional Magnetic Resonance Imaging of the Brain] Software Library (FSL) (https://fsl.fmrib.ox.ac.uk/fsl/fslwiki/FSL, accessed on 19 March 2024), a comprehensive library of analysis tools for FMRI, MRI and DTI brain imaging data (fsl.fmrib.ox.ac.uk/fsl/fslwiki/), and MRtrix (www.mrtrix.org) software packages. After eddy-correction of diffusion data, fractional anisotropy (FA) maps were calculated for each enrolled subject using FSL. In each healthy control, we then used constrained spherical deconvolution tractography (CSD) to identify the arcuate fasciculus and the corticospinal tract in the native diffusion space, as previously described [21]. The pipeline included (i) the identification of the seed and target regions in the Montreal Neurological Institute (MNI) templates, using the same regions described by Chen and colleagues for the arcuate fasciculus [22] and the left primary motor cortex and the left internal capsule and right medulla oblongata for the corticospinal tract; (ii) non-linear translation of the seed and target regions from the MNI space to each subject native diffusion space using the software package nifty-reg (https://github.com/KCL-BMEIS/niftyreg, accessed on 19 March 2024); (iii) probabilistic CSD tractography in the native diffusion space for each healthy subject using the masks defined in (ii); (iv) non-linear translation of the tractography results in the MNI space using nifty-reg; and (v) creation of population-based maps in the MNI space of the left arcuate fasciculus and the left corticospinal tract, including only those voxels identified in at least 5 controls out of 10 in each mask.

The masks thus obtained were then translated in the native diffusion space of each control and of the two patients using a non-linear transformation. After visual inspection, the mean FA values were extracted for all subjects.

### 2.2. Patients

Both patients are affected by X-linked creatine transporter deficiency. In both cases, the diagnosis was made by demonstrating a lack of brain creatine using magnetic resonance spectroscopy and presence of a known defective creatine transporter gene. Both patients had right hand dominance.

Patient 1 was, at the time of this investigation, 28 years-old (y.o.), and had a moderately severe mental retardation and speech impairment characterized by severe difficulty in word retrieval. He had to be institutionalized soon after this investigation because, when his grandmother died, his other family members were not able to take care of him due to the severity of his dependency.

Patient 2 was, at the time of this investigation, 18 y.o., and had a mild mental retardation that allowed him to attend school with sufficient proficiency. His verbal impairment was mild.

Table 1 summarizes the clinical data of both patients.

### 2.3. Statistics

We used the Mann–Whitney test to compare the patients’ data with the data from control subjects. We chose this test because it is a non-parametric test, which is suitable for comparing small samples with each other. 

## 3. Results

**Figure 1:**
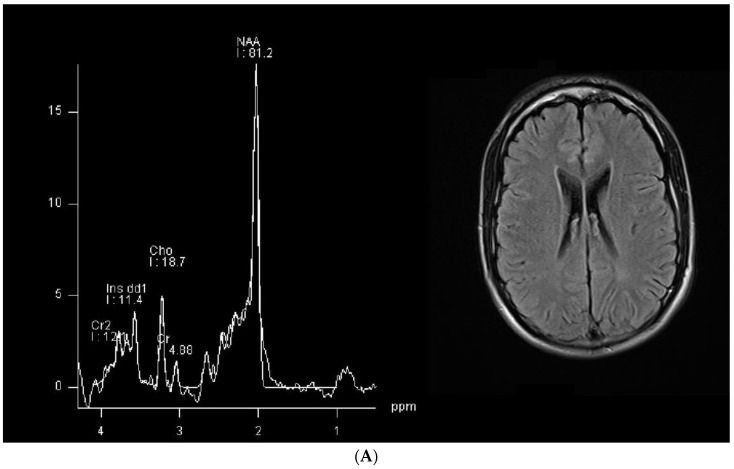

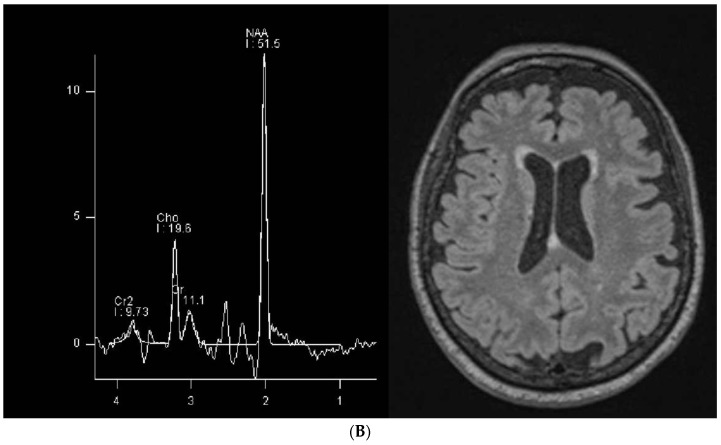
Single-voxel MR spectroscopy and a representative FLAIR axial section from our patients (patient 1 in the upper panel, marked as (**A**), and patient 2 in the bottom panel, marked as (**B**)). In the spectrogram, the peaks are plotted from right to left along the *x*-axis, and the *y*-axis (height) is the degree of chemical shift, expressed as parts per million (ppm).

The conventional MR imaging results were unremarkable for the two patients and single-voxel MRS showed minimal brain creatine, as expected (Figure 1).

**Figure 2:**
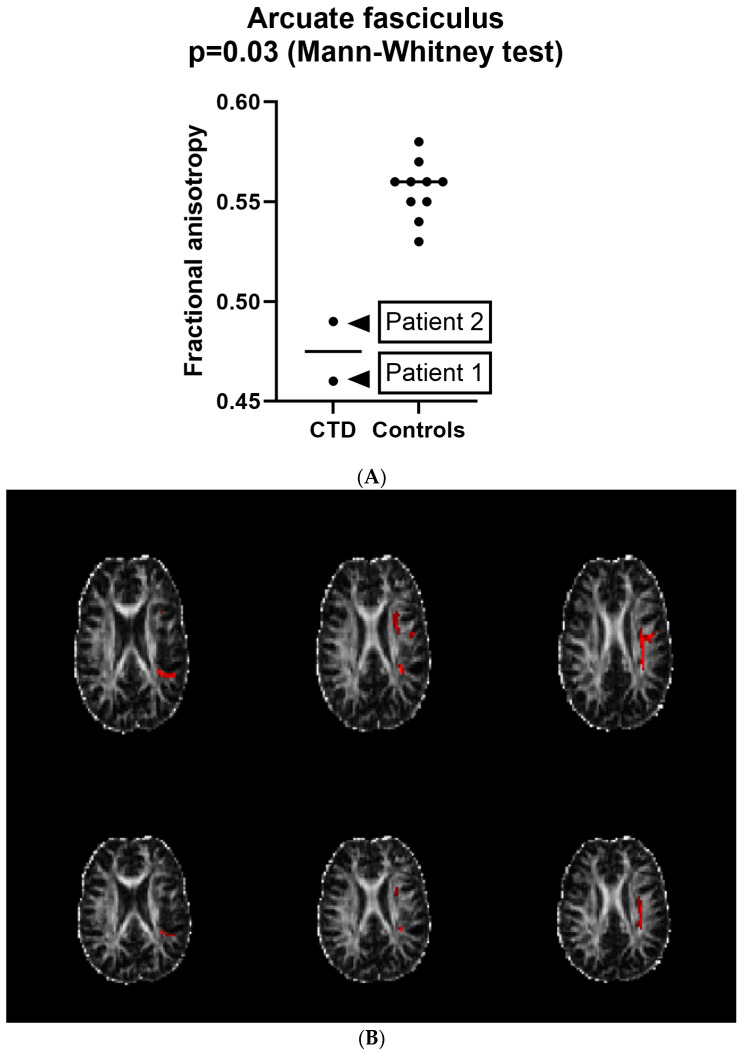
(**A**) Left arcuate fasciculus fractional anisotropy showing lower values in our two patients compared to a control group. Bars represent median values. CTD = creatine transporter deficiency. Panel (**B**) represents the tractography images, with the upper row representing patient 1 and the lower row representing patient 2.

In both patients, fractional anisotropy (FA) of the left arcuate fasciculus was reduced compared to a series of healthy controls, and the decrease was statistically significant despite the low number of observations. Moreover, there was a gross correspondence between the degree of language impairment and the degree of left arcuate fasciculus impairment. Specifically, the patient with the worst functional impairment had the lowest FA value, and vice versa (Figure 2; panel A provides the FA values of the tract and panel B is a visual representation of the tract in each individual patient).

**Figure 3:**
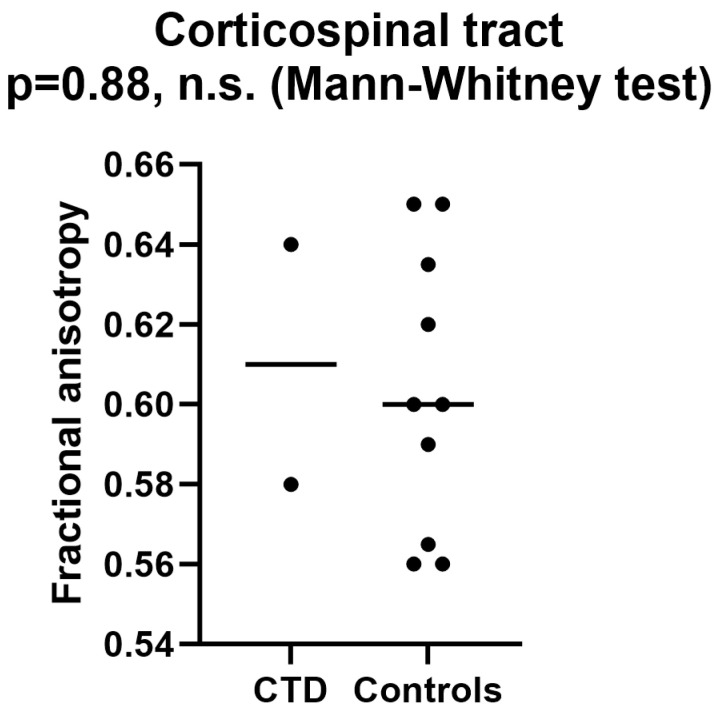
Pyramidal tract fractional anisotropy showed similar values in our two patients compared to a control group. Bars represent median values. CTD = creatine transporter deficiency.

In contrast, in both patients, the fractional anisotropy (FA) results of the pyramidal (corticospinal) tract were not different from a series of healthy controls (Figure 3).

## 4. Discussion

Human diseases involving a lack of brain creatine provide a unique opportunity to study the roles of creatine in the nervous system in humans. Two such diseases are caused by inborn deficits of creatine synthesis: L-arginine:glycine amidinotransferase (AGAT) deficiency [23] and guanidinoacetate methyltransferase (GAMT) deficiency [24]. A third one is creatine transporter (CRT) deficiency, a condition where the transporter that transport creatine across the blood–brain barrier and across cell plasma membranes is missing [8]. It is generally believed that creatine has a functional role, limited to “shuttling” ATP from the mitochondrion to the cytoplasmic sites where it is utilized [25] and to providing (through its derivative phosphocreatine) a “ready to use” energy reserve to exploit in cases of increased energy demand or of oxygen/glucose deprivation [3]. Accordingly, the administration of exogenous creatine to patients suffering from AGAT or GAMT deficiency is able to reverse, at least to a certain extent, the clinical symptoms, thus reinforcing the view that creatine’s effects are limited to its role in metabolism [26]. However, it is apparent that exogenous creatine is only able to partially reverse the symptoms of innate creatine deficiency [27,28]. By contrast, the administration of creatine at the neonatal stage completely prevented the pathological phenotype in one baby affected by AGAT deficiency [29]. These findings strongly suggest that the harmful effects of creatine deficiency in the brain are not limited to altered metabolism but may involve some kind of structural change in the brain anatomy that the administration of exogenous creatine at older ages cannot fully reverse. Supporting this view, several authors have reported that creatine is involved in neuronal differentiation [12,30,31]. Moreover, the lack of creatine may be the reason for impaired axonal development in hyperammonemia [32]. Furthermore, the preclinical data show that creatine improves oligodendrocyte survival after myelin damage [33] and that a lack of cerebral creatine (obtained through prevention of its endogenous synthesis by inactivation of the enzyme guanidinoacetate methyl transferase) disrupts myelination [34,35]. These findings strongly suggest that creatine deficiency may cause its harmful effects not only by perturbing energy metabolism, but also by preventing the correct development of at least some parts of the nervous system or of some cell populations. In creatine transporter deficiency, language impairment is particularly severe, to the extent that two-thirds of patients cannot speak in sentences [14]. The arcuate fasciculus is a white matter bundle that connects the two main language areas of the brain, namely Wernicke’s and Broca’s areas. Magnetic Resonance (MR) tractography has demonstrated that this fiber bundle is very often involved in developmental diseases characterized by language disorders [18]. Thus, we decided to use MR tractography to investigate the arcuate fasciculus in two cases of creatine transporter deficiency. We found it to be significantly altered compared to normal controls, and more altered in the patient with the more severe condition (Figure 2). Importantly, the conventional MRI results were normal in both cases (Figure 1). To the best of our knowledge, this is the first report of structural abnormalities in language white matter tracts in subjects with creatine deficiency. It suggests a possible region-specific impact of creatine deficiency in the development of white matter architecture. Although it is known that creatine administration in creatine deficiency syndromes is more effective when it is started early in life, the explanation for this fact has not been found. Our findings suggest that it may be because creatine plays a fundamental role not only in energy metabolism, but also in neuronal and brain development.

Interestingly, hypotrophy of the left arcuate fasciculus has been demonstrated to occur in primary progressive aphasia, a condition in elderly people where their ability to speak is progressively eroded in a dementia-like fashion [36]. These authors reported a decrease in the N-acetyl aspartate to creatine ratio in an area corresponding to the left arcuate fasciculus, and they interpreted this finding as a marker of neuronal dysfunction in the area under scrutiny. This further adds to the data suggesting that disruption of the left arcuate fasciculus is a cause of language impairment.

To the best of our knowledge, in the creatine transporter deficiency literature this is the first report of a selective impairment of a white matter bundle and of a localized cerebral impairment that correlates with functional symptoms. However, white matter abnormalities have been detected in this disease before; Heussinger et al. found reduced white matter volume in one patient, and they hypothesized that such atrophy may be related to mental development [15]. Our data confirm those reports by showing hypotrophy of the left arcuate fasciculus; however, they are more specific, indicating an impairment of the left arcuate fasciculus. Moreover, Anselm et al. [37] reported a thinning of the corpus callosum in two unrelated boys affected by creatine transporter deficiency with a severe functional impairment.

By contrast, it is important to note that severe brain damage in creatine transporter deficiency may occur in the context of a brain which appears fully normal in conventional magnetic resonance imaging (MRI) examinations. As already mentioned, the extensive review by van de Kamp and others of 101 affected males reported MRI alterations in only 53 out of 76 patients in whom MRI data were available (70%); it should therefore be concluded that in the remaining 23 cases (30%), the MRI results were normal. Even more clearly, Morey et al. reported the unusual but instructive case of a heterozygous female patient who was affected by creatine transporter deficiency and displayed severe symptoms despite normal conventional MRI results [38]. It is then noteworthy that in our two cases, abnormal tractography findings were observed in the face of normal conventional MRI results (Figure 1).

It is relevant to note that our results showed that underdevelopment of white matter tracts in creatine transporter deficiency is selective, involving specific white matter pathways. In fact, we found an underdevelopment of the arcuate fasciculus but not of the pyramidal tract (see Figure 2 and Figure 3). This opens the possibility that the underdevelopment we found may be not the cause, but the result, of impaired language development. In other words, the arcuate fasciculus may be underdeveloped in CTD patients not primarily because of the lack of brain creatine but because of reduced usage or development of the language areas it connects. This latter hypothesis may be supported by the fact that the pyramidal tract in our two patients was comparable to that of control people (Figure 3).

While additional research is necessary, we must consider that the underdevelopment of the arcuate fasciculus (and possibly of other white matter tracts that are relevant for intellectual development) can be caused by either a lack of creatine affecting axonal myelination [33,34,35] or by the underdevelopment or underuse of cortical areas where white matter tracts originate or terminate. The relative importance of these factors remains an area to be studied in the future. An additional aspect to be considered is that creatine deficiency may affect not only the development of white matter bundles such as the arcuate fasciculus but also cortical GABAergic interneurons [31] and synaptic spines [7]; thus, it may cause malfunction of cortical areas which in turn may lead to the underdevelopment of the white matter bundles that originate from them.

In summary, morphological changes in the brain of CTD patients exist, and they need specialized techniques like tractography to be fully revealed. One such structural change appears to be the underdevelopment of the arcuate fasciculus and possibly other connections between cortical areas.

Further research, including confirmation of these results in a larger number of patients and in other creatine deficiency syndromes, is needed to fully understand this issue.

Last but not least, the notion that creatine deficiency may affect the development or wiring of the brain is one more reason to emphasize the need for neonatal screening of creatine deficiency syndromes, since only neonatal diagnosis may lead, in cases where creatine supplementation is efficacious, to early enough treatment to fully prevent phenotype expression [29].

## Figures and Tables

**Table 1:** Clinical characteristics of the two patients.

	Patient 1	Patient 2
Age	28 years old	18 years old
Social environment	He lived with his family at the time of this investigation; he had to be admitted to institutional care shortly afterwards upon the death of his grandmother who cared for him.	He lived with his family, and he still does so.
Speech	He only speaks a few words with semantic meanings; verbal understanding is spared.	Some difficulty in finding words but information is conveyed and his vocabulary has a fair amount of complexity.
Cognitive state	Severe cognitive impairment, lack of attention with only occasional collaboration; he is often agitated, especially in unfamiliar situations.	Cognitive impairment but he shows a fair amount of attention and concentration; behavior is not grossly abnormal.
Seizures	Seizures in the past, not in the past two years.	Seizures up to the age of 6, but no seizures since then.
Neurological examination	No focal lesion signs, global motor impairment, fatuity, hyperkinesia, mild axial hypotonia, internal rotation of the feet. Right hand dominance.	Normal neurological examination except for slight impairment in fine motor movements of the hand fingers, bilaterally. Right hand dominance.
Drug therapy	Valproic acid 500 mg t.i.d. Phenobarbital 100 mg in the evening Clonazepam 1 + 1 + 2 mg/day L-arginine 14.94 g/day Levomepromazine 25 mg in case of psychomotor agitation	Valproic acid slow release 300 mg in the morning, 450 mg in the evening L-arginine 13.28 g/day

## Data Availability

The original contributions presented in the study are included in the article; further inquiries can be directed to the corresponding author.
